# Supplementary material for: Circulating fatty acids and risk of hepatocellular carcinoma and chronic liver disease mortality in the UK Biobank
Source: Nat Commun. 2024 May 2;15:3707. doi: 10.1038/s41467-024-47960-8 (PMC11065883; doi:10.1038/s41467-024-47960-8)
Supplement: Supplementary file 1 — Supplementary Information [file 41467_2024_47960_MOESM1_ESM.pdf]

Supplementary information for

Circulating fatty acids and risk of hepatocellular carcinoma and chronic liver disease mortality in the UK Biobank

Zhening Liu, Hangkai Huang, Jiarong Xie, Yingying Xu, Chengfu Xu

***Corresponding authors:**

Chengfu Xu, MD, Department of Gastroenterology, the First Affiliated Hospital, Zhejiang University School of Medicine. No. 79 Qingchun Road, Hangzhou 310003, China. Phone: 0086-571-87236863; E-mail: xiaofu@zju.edu.cn

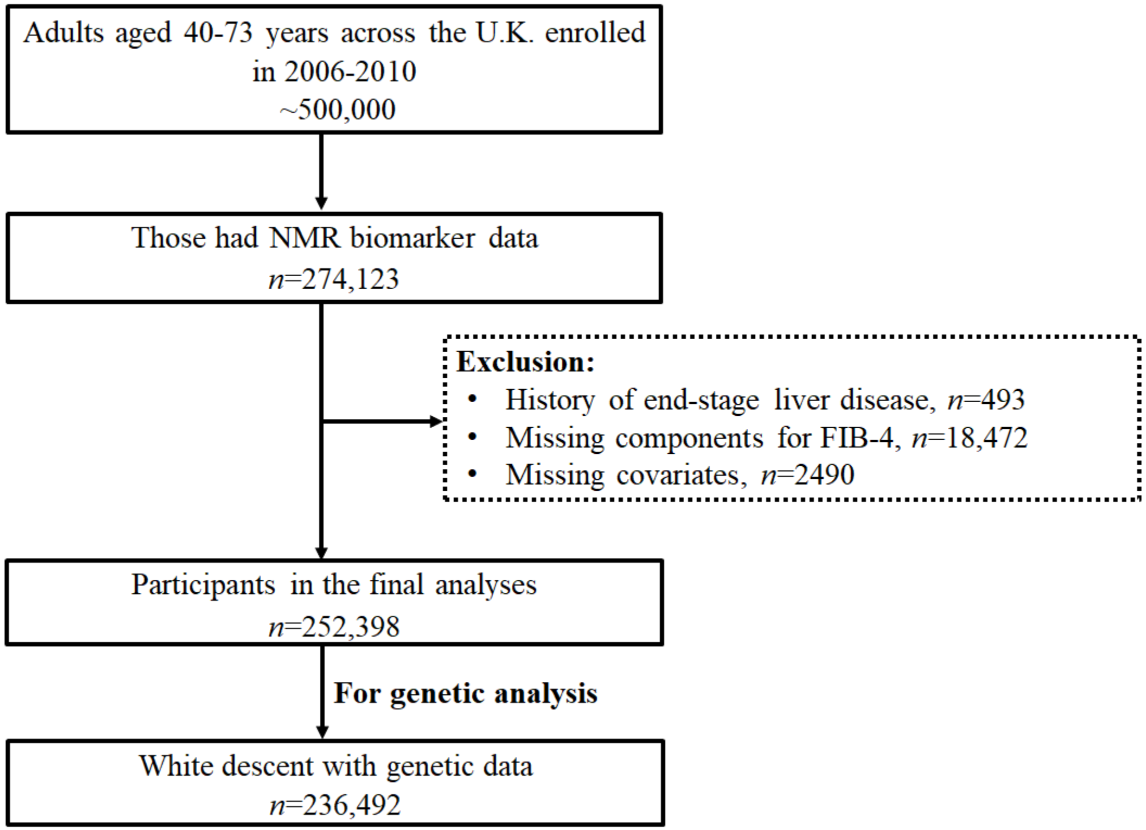

Supplementary Figure 1. Flow chart of the study design and analytical strategy

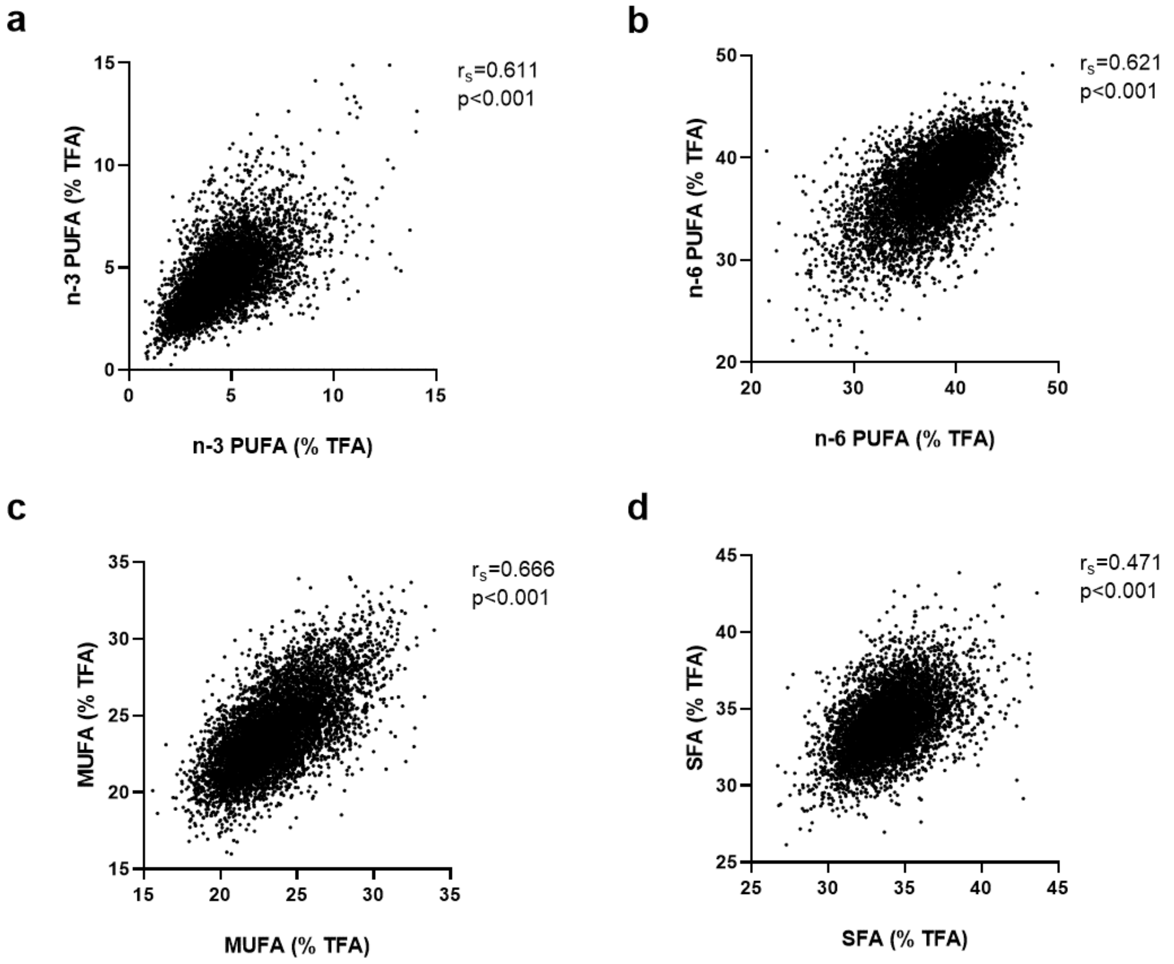

Supplementary Figure 2. Correlation of baseline fatty acids levels (2006–10) and first repeat assessment visit fatty acids levels (2012–13)

(a) Correlation of baseline n-3 PUFA level and first repeat assessment visit n-3 PUFA level.
(b) Correlation of baseline n-6 PUFA level and first repeat assessment visit n-6 PUFA level.
(c) Correlation of baseline MUFA level and first repeat assessment visit MUFA level.
(d) Correlation of baseline SFA level and first repeat assessment visit SFA level.

Source data are provided as a Source Data file.

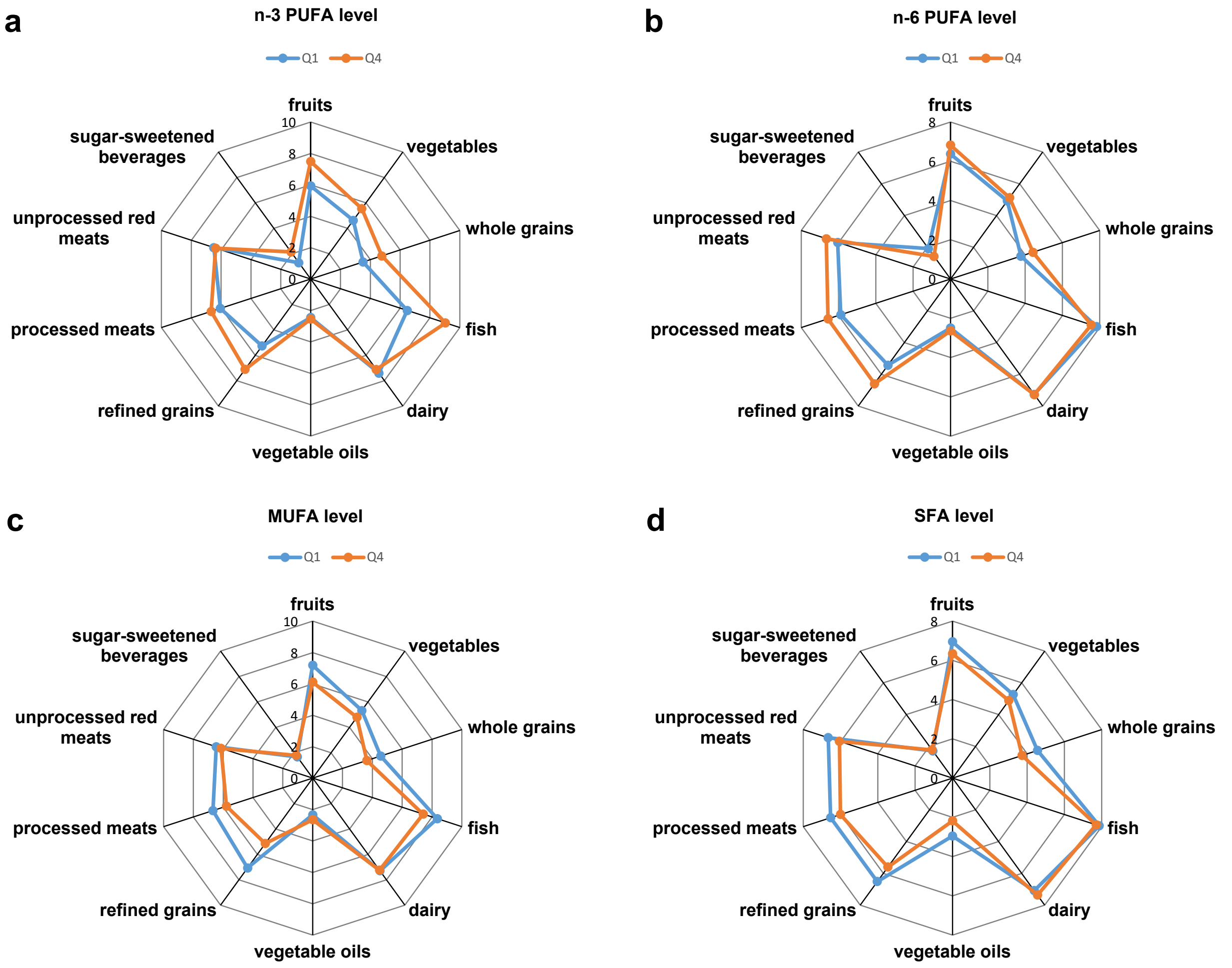

Supplementary Figure 3. Scores of individual dietary components for specific fatty acids levels

(a) Scores of individual dietary components for n-3 PUFA level.
(b) Scores of individual dietary components for n-6 PUFA level.
(c) Scores of individual dietary components for MUFA level.
(d) Scores of individual dietary components for SFA level.

Each dietary component was scored from 0 (unhealthiest) to 10 (healthiest) points. A higher score represents higher intake of vegetables, fruits, fish, dairy, whole grains, and vegetable oils or lower intake of refined grains, processed meats, unprocessed red meats, and sugar-sweetened beverages. Only the scores for Q1 and Q4 are shown here.

Source data are provided as a Source Data file.

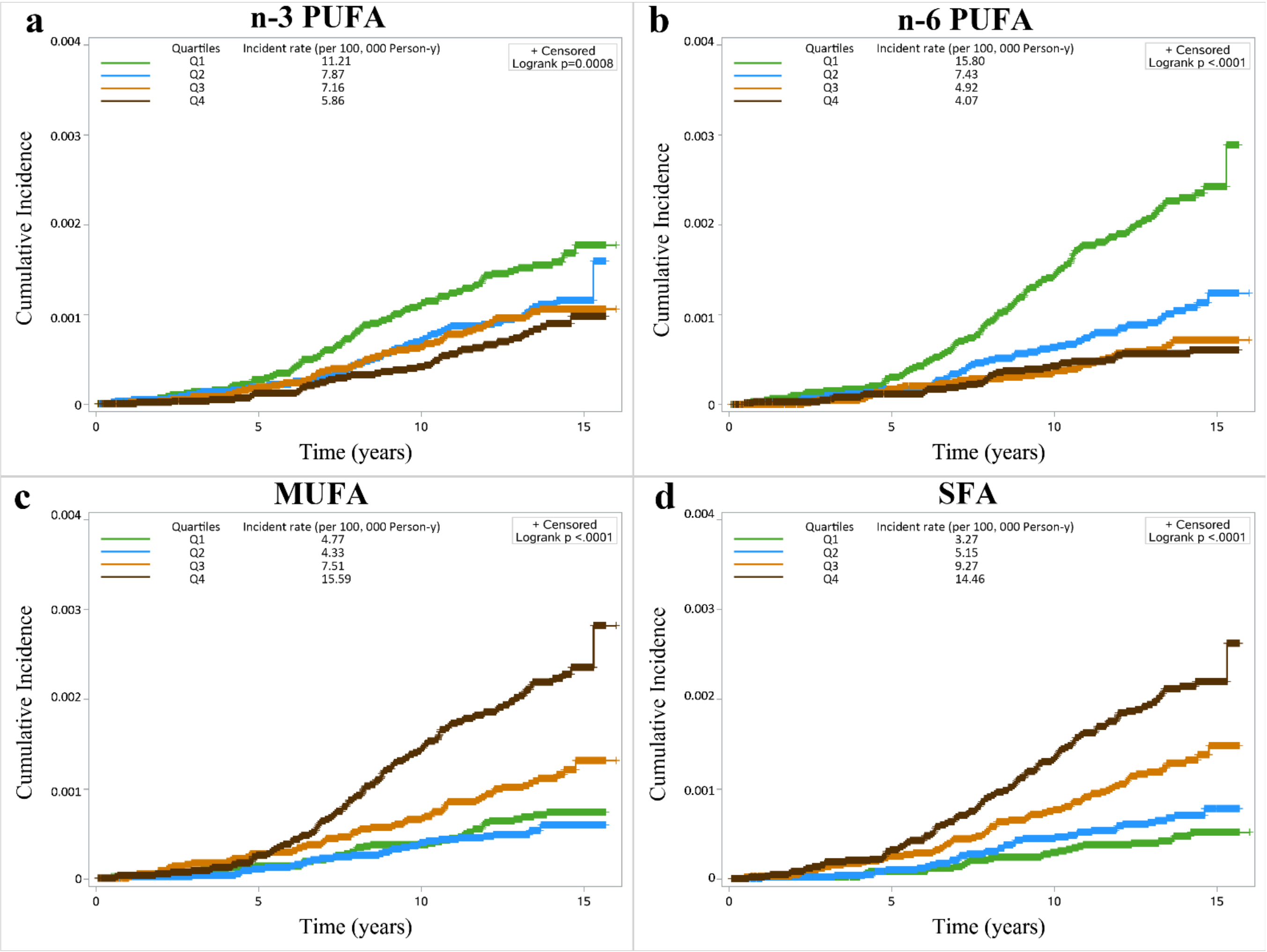

Supplementary Figure 4. Kaplan-Meier survival estimates according to specific fatty acids levels for the probability of incident HCC

(a) Kaplan-Meier survival estimates according to n-3 PUFA level for the probability of incident HCC.
(b) Kaplan-Meier survival estimates according to n-6 PUFA level for the probability of incident HCC.
(c) Kaplan-Meier survival estimates according to MUFA level for the probability of incident HCC.
(d) Kaplan-Meier survival estimates according to SFA level for the probability of incident HCC.

Source data are provided as a Source Data file.

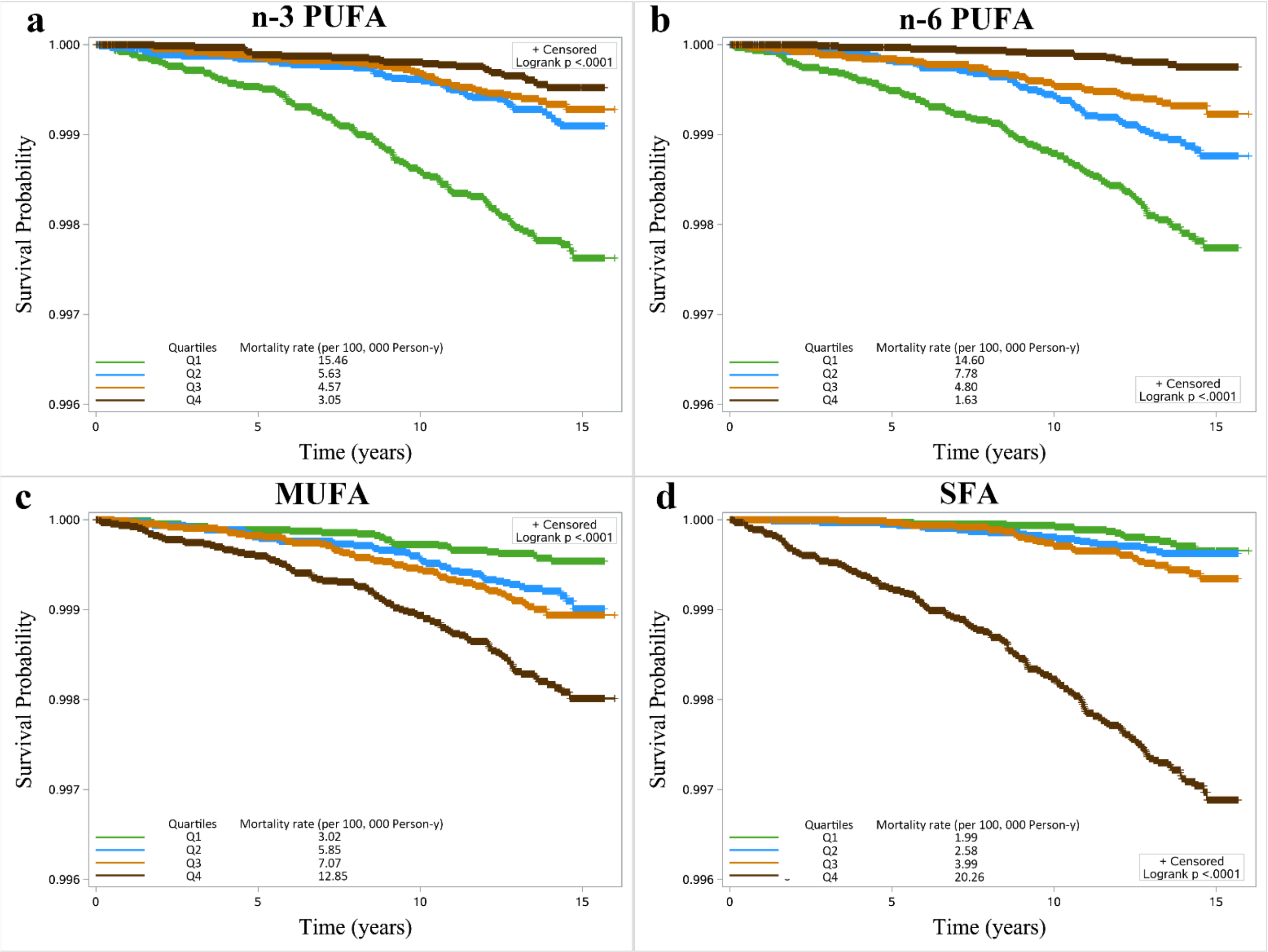

Supplementary Figure 5. Kaplan-Meier survival estimates according to specific fatty acids levels for the probability of CLD mortality

(a) Kaplan-Meier survival estimates according to n-3 PUFA level for the probability of CLD mortality.
(b) Kaplan-Meier survival estimates according to n-6 PUFA level for the probability of CLD mortality.
(c) Kaplan-Meier survival estimates according to MUFA level for the probability of CLD mortality.
(d) Kaplan-Meier survival estimates according to SFA level for the probability of CLD mortality.

Source data are provided as a Source Data file.

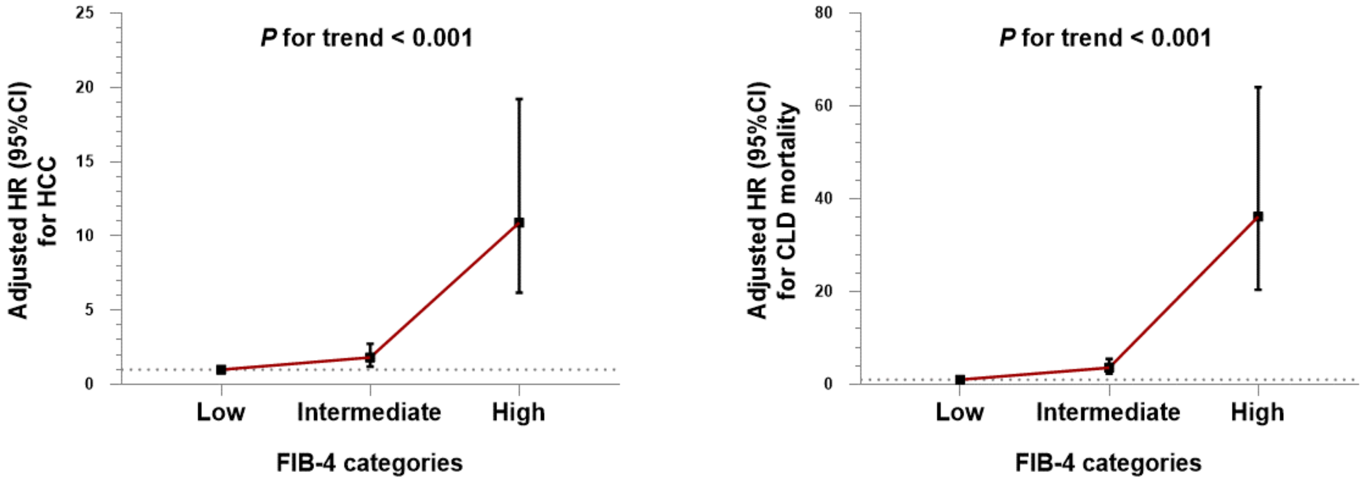

Supplementary Figure 6. Associations of FIB-4 categories with the risk of incident HCC and CLD mortality

Multivariable Cox proportional hazard model was used. Model was adjusted for age, sex, ethnicity, BMI, waist circumference, Townsend deprivation index, education level, household income, self-reported smoking status, self-reported frequency of alcohol intake, physical activity, diet quality score, baseline hypertension, baseline diabetes, baseline dyslipidemia, total cholesterol level, triglycerides level, total fatty acids level, serum ALT level, serum AST level, and blood platelet count. Data are presented as HRs and 95% CI.

Source data are provided as a Source Data file.

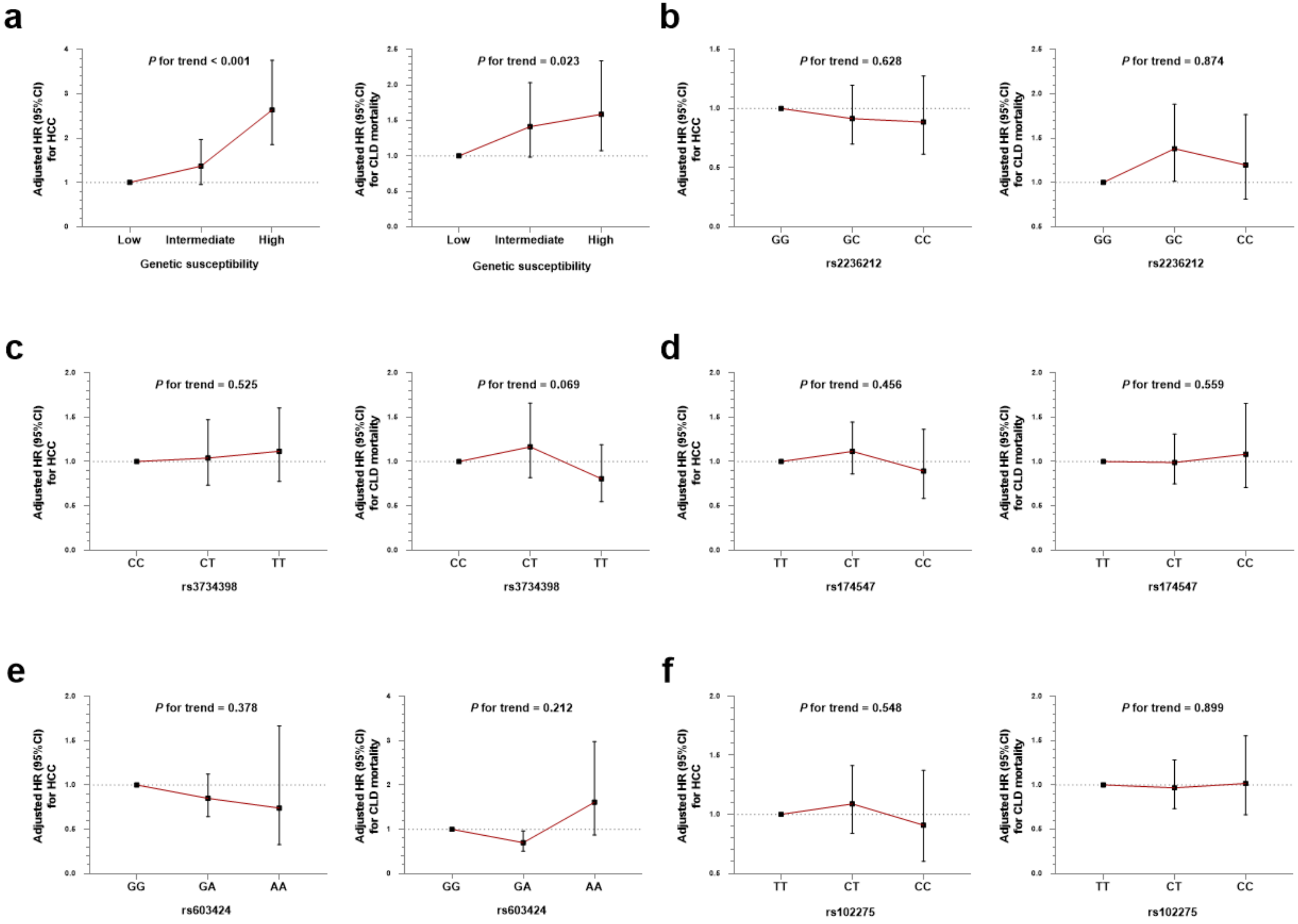
a
P for trend < 0.001
Adjusted HR (95% CI) for HCC
Low
Intermediate
High
Genetic susceptibility
P for trend = 0.023
Adjusted HR (95% CI) for CLD mortality
Low
Intermediate
High
Genetic susceptibility
b
P for trend = 0.628
Adjusted HR (95% CI) for HCC
GG
GC
CC
rs2236212
P for trend = 0.874
Adjusted HR (95% CI) for CLD mortality
GG
GC
CC
rs2236212
c
P for trend = 0.525
Adjusted HR (95% CI) for HCC
CC
CT
TT
rs3734398
P for trend = 0.069
Adjusted HR (95% CI) for CLD mortality
CC
CT
TT
rs3734398
d
P for trend = 0.456
Adjusted HR (95% CI) for HCC
TT
CT
CC
rs174547
P for trend = 0.559
Adjusted HR (95% CI) for CLD mortality
TT
CT
CC
rs174547
e
P for trend = 0.378
Adjusted HR (95% CI) for HCC
GG
GA
AA
rs603424
P for trend = 0.212
Adjusted HR (95% CI) for CLD mortality
GG
GA
AA
rs603424
f
P for trend = 0.548
Adjusted HR (95% CI) for HCC
TT
CT
CC
rs102275
P for trend = 0.899
Adjusted HR (95% CI) for CLD mortality
TT
CT
CC
rs102275

Supplementary Figure 7. Associations of genetic susceptibility and specific SNPs with the risk of incident HCC and CLD mortality

(a) The association of PRS with the risk of incident HCC and CLD mortality.
(b) The association of rs2236212 genotype with the risk of incident HCC and CLD mortality.
(c) The association of rs3734398 genotype with the risk of incident HCC and CLD mortality.
(d) The association of rs174547 genotype with the risk of incident HCC and CLD mortality.
(e) The association of rs603424 genotype with the risk of incident HCC and CLD mortality.
(f) The association of rs102275 genotype with the risk of incident HCC and CLD mortality.

Multivariable Cox proportional hazard model was used. Model was adjusted for age, sex, ethnicity, BMI, waist circumference, Townsend deprivation index, education level, household income, self-reported smoking status, self-reported frequency of alcohol intake, physical activity, diet quality score, baseline hypertension, baseline diabetes, baseline dyslipidemia, total cholesterol level, triglycerides level, total fatty acids level, serum ALT level, serum AST level, and blood platelet count. Data are presented as HRs and 95% CI.

Source data are provided as a Source Data file.

Supplementary Table 1. Criteria for the end stage liver disease

ICD-10 Code	Description
**K74.6**	Other and unspecified cirrhosis of liver
**K76.6**	Portal hypertension
**K76.7**	Hepatorenal syndrome
**I85.0**	Oesophageal varices with bleeding
**I85.9**	Oesophageal varices without bleeding
**I86.4**	Gastric varices
**I98.2**	Oesophageal varices in diseases classified elsewhere
**I98.3**	Oesophageal varices with bleeding in diseases classified elsewhere
**R18**	Ascites
**Z94.4**	Liver transplant
**C22.0**	Liver cell carcinoma

Supplementary Table 2. Components and scaling methods of diet quality score used in the UK Biobank study

Components	Data field IDs	Amount per serving	Criteria for maximum score (10)	Criteria for minimum score (0)
Fruit	1309 (pieces fresh fruit/day)<br>1319 (pieces dried fruit/day)	1309 – 1 piece<br>1319 – 5 pieces	≥3 servings/day	0 servings/day
Vegetable	1289 (tablespoons cooked vegetables/day)<br>1299 (salad/raw vegetables/day)	3 heaped tablespoons	≥3 servings/day	0 servings/day
Whole grains	1438, 1448 (whole meal/whole grain bread slices/week)<br>1458, 1468 (bran/oat/muesli cereal bowls/week)	1438/1448 – 1 slice/day<br>1458/1468 – 1 bowl/day	≥3 servings/day	0 servings/day
Fish	1329 (oily fish/week)<br>1339 (non-oily fish/week)	Once/week	≥2 servings/week	0 servings/week
Dairy	1408 (cheese/week)<br>1418 (milk type)	1408 – 1 piece/day<br>1418 – 1 glass/day if consumption of any type of milk	≥2 servings/day	0 servings/day
Vegetable oils	1428 (Flora Pro-Active/Benecol spread)<br>2654 (Flora Pro-Active/Benecol, soft margarine -, olive oil based -, polyunsaturated/sunflower oil based -, other low/reduced fat spread)<br>1438 (bread slices/week)	1 serving/day if in combination with eating at least 2 slices of bread (ID 1438)	≥2 servings/day	0 servings/day
Refined grains	1438<br>1448 (white, brown, other bread slices/week)<br>1458	1438/1448 – 1 slice/day 1458/1468 – 1bowl/day	0 servings/day	>2 servings/day

	1468 (biscuit, other cereals/week)			
Processed meats	1349 (processed meat/week or daily)<br>3680(age when last ate meat)	1349 – 1 piece/day<br>3680 – 0 pieces/day if indicated having never eaten meat	0 serving/week	>1 serving/week
Unprocessedred meats	1369 (beef/week or day)<br>1379 (lamb or mutton/week or day)<br>1389(pork/week or day)<br>3680 (age when last ate meat)	1359-1389 – once/week<br>3680 – 0 pieces/day if indicated having never eaten meat	0 serving/week	>2 serving/week
Sugar-sweetened beverages	6144 (never consumes drinks containingsugar)	0 servings	Don't drink	Drink

component score = [maximum score / (A_{max} - A_{min})]×(X - A_{min}) for adequacy components;
component score = [maximum score - maximum score / (A_{max} - A_{min})]×(X - A_{min}) for moderate components (refined grains, processed meat, and unprocessed red meat); A_{max} is maximum amount of the component corresponding to the recommended intake; A_{min} is minimum amount of the component corresponding to the recommended intake; X is amount consumed by the individual. The total diet quality score was the sum of all the diet component scores and ranged from 0 to 100.

Reference: Zhuang P, Liu X, Li Y, Wan X, Wu Y, Wu F, et al. Effect of Diet Quality and Genetic Predisposition on Hemoglobin A and Type 2 Diabetes Risk: Gene-Diet Interaction Analysis of 357,419 Individuals. Diabetes Care. 2021;44(11):2470-9.

Supplementary Table 3. Characteristics of 5 liver-associated SNPs in the UK Biobank

rsID	Chromosome	Effect allele	Beta
rs1260326	2	T	0.065
rs72613567	4	TA	-0.361
rs58542926	19	T	0.274
rs641738	19	T	0.063
rs738409	22	G	0.266

Supplementary Table 4. SNPs associated with circulating individual fatty acids.

Type of fatty acids	Fatty acid	SNP	Chr	Nearby gene	EA	NEA	% Variance explained	Association with fatty acids		
								**Beta**	**SE**	***P***
n-3 PUFA	ALA	rs174547	11	FADS1	C	T	1.0	0.02	0.001	3.5×10^{-64}
n-3 PUFA	DHA	rs2236212	6	ELOVL2	G	C	0.7	0.11	0.014	1.3×10^{-15}
n-3 PUFA	DPA	rs3734398	6	ELOVL2	C	T	2.7	0.04	0.003	9.7×10^{-43}
n-3 PUFA	DPA	rs174547	11	FADS1	T	C	8.4	0.08	0.003	3.8×10^{-154}
n-6 PUFA	LA	rs174547	11	FADS1	C	T	7.6-18.1	1.47	0.050	5.0×10^{-274}
n-6 PUFA	AA	rs174547	11	FADS1	T	C	3.7-37.6	1.69	0.020	3.3×10^{-971}
MUFA	POA	rs603424	10	SCD/PKD2L1	G	A	0.3-1.6	0.03	0.004	5.7×10^{-15}
MUFA	POA	rs102275	11	FADS1/2	C	T	0.15-1.0	0.02	0.003	6.6×10^{-13}
MUFA	OA	rs102275	11	FADS1/2	C	T	0.3-2.1	0.23	0.020	2.2×10^{-32}
SFA	SA	rs102275	11	FADS1/2	T	C	0.3-1.2	0.18	0.020	1.3×10^{-20}

AA, arachidonic acid; ALA, α-linolenic acid; Chr, chromosome; DPA, docosapentaenoic acid; EA, effect allele; EPA, eicosapentaenoic acid; LA, linoleic acid; NEA, non-effect allele; OA, oleic acid; PA, palmitic acid; POA, palmitoleic acid; SA, stearic acid.

Supplementary Table 5. Population summary characteristics

Variables	n-3 PUFA (% TFA)				*P* value	SFA (% TFA)				*P* value
	Q1 (<3.3)	Q2 (3.3–4.2)	Q3 (4.2–5.2)	Q4 (>5.2)		Q1 (<32.7)	Q2 (32.7–33.9)	Q3 (33.9–35.2)	Q4 (>35.2)	
Male (%)	54.2	49.6	44.0	36.6	<0.001	48.4	41.6	42.5	51.8	<0.001
Age (years)	54.5±8.2	55.8±8.1	57.2±7.9	58.8±7.4	<0.001	55.8±8.3	56.3±8.1	56.8±8	57.3±7.8	<0.001
Ethnicity (%)					<0.001					<0.001
White	94.9	95.5	95.4	93.9		89.9	95.6	96.8	97.4	
Asian	2.9	2.1	1.6	1.5		4.7	1.5	1.0	0.9	
Black	0.5	0.8	1.4	2.7		2.7	1.3	0.8	0.5	
Others	1.7	1.6	1.6	1.9		2.7	1.6	1.4	1.1	
Townsend deprivation index	-0.9±3.2	-1.3±3.1	-1.6±3	-1.7±2.9	<0.001	-1.2±3.2	-1.5±3.0	-1.5±3.0	-1.3±3.1	<0.001
College or university degree (%)	28.8	30.5	32.6	35.2	<0.001	35.2	32.0	31.1	28.8	<0.001
Household income (£)					<0.001					<0.001
<18,000	21.8	19.6	19.1	18.6		19.7	18.9	19.2	21.3	
18,000 to 30,999	22.5	21.6	21.8	22.6		21.7	22.0	21.9	22.9	
31,000 to 51,999	23.0	23.0	22.0	20.9		22.2	22.7	22.2	21.8	
52,000 to 100,000	16.1	17.8	17.6	16.6		17.3	17.2	17.4	16.2	
>100,000	3.2	4.2	4.8	5.3		4.4	4.6	4.6	3.9	
Physical activity (%)					<0.001					<0.001
Inadequate	15.8	15.7	15.4	13.5		14.5	14.2	14.8	16.9	
Moderate	37.9	39.8	41.5	43.1		42.1	40.9	40.3	39.0	
Vigorous	26.2	25.2	24.4	25.0		25.2	25.7	25.4	24.6	
Smoking status (%)					<0.001					<0.001

Never	52.0	53.8	54.9	57.5		60.1	57.2	54.1	46.9	
Previous	31.3	34.7	36.7	36.9		31.7	33.6	35.3	39.1	
Current	16.7	11.5	8.4	5.6		8.3	9.2	10.6	14.0	
Alcohol consumption (%)					<0.001					<0.001
Never or special occasions only	23.0	18.7	17.6	17.5		25.8	19.1	16.3	15.5	
1 to 3 times/month	12.7	11.7	10.5	9.5		14.0	11.7	10.2	8.6	
1 to 4 times/week	47.1	50.1	50.5	50.2		48.7	52.0	50.8	46.5	
Daily or almost daily	17.2	19.5	21.4	22.8		11.6	17.2	22.7	29.4	
Waist circumference (cm)	91.9±14.2	91.5±13.5	90.1±13	87.5±12.5	<0.001	88.5±12.5	88.6±13.2	89.8±13.6	94.1±13.6	<0.001
Body mass index (kg/m^2)	27.8±5.3	27.8±4.8	27.4±4.6	26.7±4.2	<0.001	26.7±4.4	27.1±4.7	27.4±4.8	28.5±4.9	<0.001
Hypertension (%)	52.9	55.3	56.4	56.6	<0.001	48.5	51.3	55.9	65.6	<0.001
Diabetes (%)	5.7	6.1	6.5	5.6	<0.001	6.1	4.7	5.2	8.0	<0.001
Dyslipidemia (%)	39.0	43.3	42.8	35.3	<0.001	26.1	29.0	38.7	66.7	<0.001
Platelet (10^9/L)	256.5±61.6	254.6±61.0	253.3±59.0	249.3±58.0	<0.001	249±59.6	253.5±59.0	255.7±59.4	255.3±61.6	<0.001
Alanine aminotransferase (U/L)	23.7±14.8	24.0±14.5	23.7±14.2	23.0±13.3	<0.001	21.5±11.6	22.0±12.7	23.3±14.0	27.6±17.1	<0.001
Aspartate aminotransferase (U/L)	26.4±11.3	26.2±10.1	26.1±10.1	26.2±9.5	0.002	25.3±8.2	25.4±8.7	26.0±9.8	28.3±13.3	<0.001
Total cholesterol (mmol/L)	4.5±0.9	4.6±0.9	4.7±1.0	4.8±1.0	<0.001	4.4±0.9	4.6±0.9	4.7±0.9	4.8±1.0	<0.001
Triglycerides (mmol/L)	1.3±0.6	1.4±0.6	1.4±0.6	1.3±0.6	<0.001	1.1±0.4	1.2±0.5	1.3±0.5	1.7±0.7	0.004
Total fatty acid (mmol/L)	11.6±2.4	12.1±2.4	12.3±2.4	12.2±2.4	<0.001	11.1±2.1	11.6±2.1	12.1±2.2	13.5±2.7	<0.001
Diet quality score	49.5±12.6	52.4±12.3	55.0±12.0	58.8±11.3	<0.001	56.1±12.5	54.6±12.3	53.3±12.3	51.7±12.7	<0.001

Values are the mean (±standard deviation, SD) or percentage (%) and were examined by two-sided one-way ANOVA or chi-square test.

Supplementary Table 6. Spearman correlations between specific plasma fatty acids (% of total fatty acids).

	n-3 PUFA	n-6 PUFA	MUFA	SFA
**n-3 PUFA**	1.000	-0.137	-0.278	-0.137
**n-6 PUFA**		1.000	-0.750	-0.646
**MUFA**			1.000	0.265
**SFA**				1.000

All with *P*<0.001

Supplementary Table 7. **Associations between plasma n-3 PUFA, n-6 PUFA, MUFA, SFA levels and incident HCC risk**

Fatty acids	Range (% TFA)	Model 1		Model 2		Model 3	
		HR (95% CI)	P_{trend}	HR (95% CI)	P_{trend}	HR (95% CI)	P_{trend}
**n-3 PUFA**			<0.001		0.006		<0.001
**Q1**	<3.3	1 (Ref)		1 (Ref)		1 (Ref)	
**Q2**	3.3–4.2	0.67 (0.49–0.91)		0.70 (0.51–0.95)		0.67 (0.49–0.92)	
**Q3**	4.2–5.2	0.59 (0.42–0.81)		0.65 (0.46–0.90)		0.60 (0.43–0.83)	
**Q4**	>5.2	0.47 (0.33–0.67)		0.61 (0.42–0.88)		0.48 (0.33–0.69)	
**n-6 PUFA**			<0.001		0.001		0.018
**Q1**	<35.7	1 (Ref)		1 (Ref)		1 (Ref)	
**Q2**	35.7–38.4	0.54 (0.40–0.73)		0.56 (0.40–0.80)		0.63 (0.43–0.92)	
**Q3**	38.4–40.4	0.41 (0.29–0.57)		0.43 (0.28–0.66)		0.56 (0.35–0.89)	
**Q4**	>40.4	0.38 (0.26–0.55)		0.42 (0.26–0.69)		0.48 (0.28–0.81)	
**MUFA**			<0.001		0.018		0.037
**Q1**	<21.8	1 (Ref)		1 (Ref)		1 (Ref)	
**Q2**	21.8–23.5	0.76 (0.49–1.19)		0.64 (0.41–1.01)		0.83 (0.52–1.31)	
**Q3**	23.5–25.4	1.18 (0.80–1.75)		0.89 (0.58–1.37)		1.18 (0.75–1.86)	
**Q4**	>25.4	2.18 (1.53–3.12)		1.35 (0.82–2.20)		1.51 (0.86–2.63)	
**SFA**			<0.001		<0.001		<0.001
**Q1**	<32.7	1 (Ref)		1 (Ref)		1 (Ref)	
**Q2**	32.7–33.9	1.70 (1.06–2.74)		1.74 (1.08–2.81)		1.81 (1.12–2.93)	
**Q3**	33.9–35.2	2.95 (1.91–4.56)		2.97 (1.91–4.63)		3.26 (2.08–5.10)	
**Q4**	>35.2	4.08 (2.69–6.17)		3.76 (2.42–5.84)		3.55 (2.25–5.61)	

Multivariable Cox proportional hazard models were used: Model 1 was adjusted for age, sex, and ethnicity. Model 2 was adjusted for model 1 plus BMI, waist circumference, Townsend deprivation index, education level, household income, self-reported smoking status, self-reported frequency of alcohol intake, physical activity, diet quality score, baseline hypertension, baseline diabetes, and baseline dyslipidemia. Model 3 was adjusted for model 2 plus total cholesterol level, triglycerides level, total fatty acids level, serum ALT level, serum AST level, and blood platelet count.

Supplementary Table 8. Associations between plasma LA, DHA, n-6/n-3, MUFA/SFA and incident HCC risk

	Range†	Model 1		Model 2		Model 3	
		HR (95% CI)	P_{trend}	HR (95% CI)	P_{trend}	HR (95% CI)	P_{trend}
**LA (n-6)**			<0.001		0.060		0.617
**Q1**	<25.2	1 (Ref)		1 (Ref)		1 (Ref)	
**Q2**	25.2–27.5	0.50 (0.37–0.68)		0.71 (0.51–0.99)		0.97 (0.68–1.37)	
**Q3**	27.5–29.7	0.50 (0.36–0.69)		0.83 (0.58–1.20)		1.39 (0.94–2.06)	
**Q4**	>29.7	0.31 (0.21–0.47)		0.62 (0.40–0.98)		1.02 (0.62–1.65)	
**DHA (n-3)**			<0.001		0.175		0.042
**Q1**	<1.3	1 (Ref)		1 (Ref)		1 (Ref)	
**Q2**	1.3–1.7	0.77 (0.57–1.05)		0.89 (0.65–1.22)		0.87 (0.63–1.21)	
**Q3**	1.7–2.0	0.61 (0.44–0.85)		0.79 (0.56–1.13)		0.81 (0.56–1.17)	
**Q4**	>2.0	0.52 (0.36–0.74)		0.80 (0.54–1.19)		0.68 (0.45–1.03)	
**n-6/n-3**			0.027		0.040		<0.001
**Q1**	<7.2	1 (Ref)		1 (Ref)		1 (Ref)	
**Q2**	7.2–9.0	0.96 (0.68–1.35)		0.90 (0.64–1.27)		1.14 (0.81–1.62)	
**Q3**	9.0–11.4	0.94 (0.66–1.33)		0.89 (0.62–1.27)		1.21 (0.83–1.74)	
**Q4**	>11.4	1.38 (0.99–1.91)		1.33 (0.94–1.88)		1.95 (1.35–2.82)	
**MUFA/SFA**			0.009		0.079		0.008
**Q1**	<0.65	1 (Ref)		1 (Ref)		1 (Ref)	
**Q2**	0.65–0.69	0.69 (0.47–1.02)		0.56 (0.38–0.83)		0.63 (0.42–0.96)	
**Q3**	0.69–0.75	0.95 (0.67–1.36)		0.62 (0.42–0.90)		0.63 (0.42–0.94)	
**Q4**	>0.75	1.26 (0.91–1.76)		0.56 (0.38–0.84)		0.48 (0.31–0.76)	

Multivariable Cox proportional hazard models were used: Model 1 was adjusted for age, sex, and ethnicity. Model 2 was adjusted for model 1 plus BMI, waist circumference, Townsend deprivation index, education level, household income, self-reported smoking status, self-reported frequency of alcohol intake, physical activity, diet quality score, baseline hypertension, baseline diabetes, and baseline dyslipidemia. Model 3 was adjusted for model 2 plus total cholesterol level, triglycerides level, total fatty acids level, serum ALT level, serum AST level, and blood platelet count.
† The unit of LA and DHA were %TFA.

Supplementary Table 9. **Associations between plasma n-3 PUFA, n-6 PUFA, MUFA, SFA levels and CLD mortality**

**Fatty acids**	**Range (% TFA)**	**Model 1**		**Model 2**		**Model 3**	
		**HR (95% CI)**	P_{trend}	**HR (95% CI)**	P_{trend}	**HR (95% CI)**	P_{trend}
**n-3 PUFA**			<0.001		<0.001		<0.001
**Q1**	<3.3	1 (Ref)		1 (Ref)		1 (Ref)	
**Q2**	3.3–4.2	0.36 (0.26–0.51)		0.41 (0.29–0.58)		0.41 (0.29–0.58)	
**Q3**	4.2–5.2	0.30 (0.21–0.43)		0.37 (0.25–0.53)		0.29 (0.20–0.43)	
**Q4**	>5.2	0.21 (0.14–0.33)		0.30 (0.19–0.47)		0.21 (0.13–0.33)	
**n-6 PUFA**			<0.001		<0.001		<0.001
**Q1**	<35.7	1 (Ref)		1 (Ref)			
**Q2**	35.7–38.4	0.60 (0.44–0.81)		0.44 (0.31–0.63)		0.53 (0.36–0.78)	
**Q3**	38.4–40.4	0.39 (0.28–0.56)		0.28 (0.18–0.44)		0.40 (0.25–0.64)	
**Q4**	>40.4	0.13 (0.08–0.23)		0.11 (0.06–0.21)		0.15 (0.08–0.30)	
**MUFA**			<0.001		<0.001		<0.001
**Q1**	<21.8	1 (Ref)		1 (Ref)			
**Q2**	21.8–23.5	1.77 (1.10–2.85)		1.46 (0.90–2.36)		2.20 (1.33–3.63)	
**Q3**	23.5–25.4	1.94 (1.22–3.10)		1.61 (0.99–2.63)		2.44 (1.45–4.09)	
**Q4**	>25.4	3.16 (2.04–4.89)		2.61 (1.49–4.55)		3.81 (2.03–7.16)	
**SFA**			<0.001		<0.001		<0.001
**Q1**	<32.7	1 (Ref)		1 (Ref)			
**Q2**	32.7–33.9	1.38 (0.73–2.61)		1.27 (0.67–2.41)		1.32 (0.70–2.51)	
**Q3**	33.9–35.2	2.13 (1.19–3.82)		1.80 (0.99–3.25)		1.90 (1.04–3.45)	
**Q4**	>35.2	9.75 (5.90–16.11)		7.24 (4.26–12.30)		6.34 (3.68–10.92)	

Multivariable Cox proportional hazard models were used: Model 1 was adjusted for age, sex, and ethnicity. Model 2 was adjusted for model 1 plus BMI, waist circumference, Townsend deprivation index, education level, household income, self-reported smoking status, self-reported frequency of alcohol intake, physical activity, diet quality score, baseline hypertension, baseline diabetes, and baseline dyslipidemia. Model 3 was adjusted for model 2 plus total cholesterol level, triglycerides level, total fatty acids level, serum ALT level, serum AST level, and blood platelet count.

Supplementary Table 10. Associations between plasma LA, DHA, n-6/n-3, MUFA/SFA and CLD mortality

	Range†	Model 1		Model 2		Model 3	
		HR (95% CI)	P_{trend}	HR (95% CI)	P_{trend}	HR (95% CI)	P_{trend}
**LA (n-6)**			<0.001		<0.001		<0.001
**Q1**	<25.2	1 (Ref)		1 (Ref)		1 (Ref)	
**Q2**	25.2–27.5	0.44 (0.32–0.60)		0.56 (0.40–0.78)		0.50 (0.34–0.74)	
**Q3**	27.5–29.7	0.32 (0.22–0.46)		0.49 (0.33–0.72)		0.60 (0.39–0.91)	
**Q4**	>29.7	0.14 (0.09–0.24)		0.28 (0.16–0.48)		0.35 (0.20–0.63)	
**DHA (n-3)**			<0.001		<0.001		<0.001
**Q1**	<1.3	1 (Ref)		1 (Ref)		1 (Ref)	
**Q2**	1.3–1.7	0.70 (0.52–0.94)		0.75 (0.55–1.03)		0.61 (0.44–0.86)	
**Q3**	1.7–2.0	0.37 (0.25–0.54)		0.44 (0.29–0.66)		0.34 (0.23–0.52)	
**Q4**	>2.0	0.29 (0.19–0.45)		0.40 (0.25–0.64)		0.24 (0.14–0.39)	
**n-6/n-3**			<0.001		<0.001		<0.001
**Q1**	<7.2	1 (Ref)		1 (Ref)		1 (Ref)	
**Q2**	7.2–9.0	1.12 (0.74–1.70)		1.09 (0.71–1.66)		1.35 (0.88–2.07)	
**Q3**	9.0–11.4	1.22 (0.81–1.85)		1.18 (0.78–1.80)		1.79 (1.17–2.76)	
**Q4**	>11.4	2.39 (1.65–3.46)		2.18 (1.47–3.22)		3.61 (2.39–5.45)	
**MUFA/SFA**			0.312		0.002		0.005
**Q1**	<0.65	1 (Ref)		1 (Ref)		1 (Ref)	
**Q2**	0.65–0.69	0.82 (0.57–1.17)		0.75 (0.52–1.09)		0.87 (0.59–1.27)	
**Q3**	0.69–0.75	0.78 (0.54–1.12)		0.62 (0.42–0.93)		0.68 (0.45–1.03)	
**Q4**	>0.75	0.80 (0.56–1.15)		0.49 (0.32–0.76)		0.49 (0.30–0.81)	

Multivariable Cox proportional hazard models were used: Model 1 was adjusted for age, sex, and ethnicity. Model 2 was adjusted for model 1 plus BMI, waist circumference, Townsend deprivation index, education level, household income, self-reported smoking status, self-reported frequency of alcohol intake, physical activity, diet quality score, baseline hypertension, baseline diabetes, and baseline dyslipidemia. Model 3 was adjusted for model 2 plus total cholesterol level, triglycerides level, total fatty acids level, serum ALT level, serum AST level, and blood platelet count.
† The unit of LA and DHA were %TFA.

Supplementary Table 11. Associations between plasma fatty acids levels and CLD mortality according to the causes

CLD causes	HRs associated with 1-SD increment in specific plasma fatty acids	*P* value
**ALD (ICD-10 K70)**		
**n-3 PUFA**	0.47 (0.38–0.60)	<0.001
**n-6 PUFA**	0.23 (0.17–0.32)	<0.001
**MUFA**	3.08 (2.23–4.24)	<0.001
**SFA**	2.16 (1.82–2.57)	<0.001
**Fibrosis or cirrhosis (ICD-10 K74)**		
**n-3 PUFA**	0.46 (0.32–0.66)	<0.001
**n-6 PUFA**	0.45 (0.27–0.75)	0.002
**MUFA**	1.75 (1.04–2.94)	0.036
**SFA**	2.19 (1.64–2.93)	<0.001
**MASLD (ICD-10 K75.8 and K76.0)**		
**n-3 PUFA**	0.69 (0.42–1.13)	0.140
**n-6 PUFA**	0.53 (0.25–1.13)	0.099
**MUFA**	1.17 (0.55–2.50)	0.683
**SFA**	1.85 (1.18–2.89)	0.007

Multivariable Cox proportional hazard model was used. Model was adjusted for age, sex, ethnicity, BMI, waist circumference, Townsend deprivation index, education level, household income, self-reported smoking status, self-reported frequency of alcohol intake, physical activity, diet quality score, baseline hypertension, baseline diabetes, baseline dyslipidemia, total cholesterol level, triglycerides level, total fatty acids level, serum ALT level, serum AST level, and blood platelet count.

Supplementary Table 12. Sensitivity analyses of the HRs for the associations of plasma fatty acids levels with incident HCC risk

Fatty acids	Exclude the first 2 years of follow-up †	Further adjusted for lipid-lowering medication	Further adjusted for PRS	Further adjusted for FADS1/2 genotype ‡	Further adjusted for remaining plasma fatty acids §
**n-3 PUFA**					
**Q1**	1 (Ref)	1 (Ref)	1 (Ref)	1 (Ref)	1 (Ref)
**Q2**	0.65 (0.47–0.91)	0.70 (0.51–0.96)	0.68 (0.49–0.93)	0.63 (0.46–0.87)	0.72 (0.52–0.99)
**Q3**	0.60 (0.43–0.85)	0.64 (0.46–0.89)	0.59 (0.42–0.82)	0.55 (0.39–0.77)	0.67 (0.48–0.94)
**Q4**	0.48 (0.33–0.71)	0.52 (0.36–0.75)	0.48 (0.33–0.69)	0.44 (0.30–0.64)	0.57 (0.39–0.82)
**n-6 PUFA**					
**Q1**	1 (Ref)	1 (Ref)	1 (Ref)	1 (Ref)	1 (Ref)
**Q2**	0.63 (0.42–0.93)	0.62 (0.42–0.90)	0.65 (0.44–0.95)	0.63 (0.43–0.92)	0.70 (0.47–1.03)
**Q3**	0.59 (0.36–0.96)	0.54 (0.34–0.87)	0.58 (0.36–0.93)	0.57 (0.35–0.90)	0.61 (0.37–0.99)
**Q4**	0.44 (0.25–0.77)	0.46 (0.27–0.79)	0.53 (0.31–0.90)	0.49 (0.29–0.83)	0.50 (0.28–0.87)
**MUFA**					
**Q1**	1 (Ref)	1 (Ref)	1 (Ref)	1 (Ref)	1 (Ref)
**Q2**	0.83 (0.51–1.36)	0.82 (0.52–1.31)	0.82 (0.51–1.31)	0.83 (0.52–1.32)	0.85 (0.53–1.37)
**Q3**	1.20 (0.75–1.94)	1.19 (0.76–1.88)	1.14 (0.72–1.79)	1.18 (0.75–1.86)	1.21 (0.76–1.95)
**Q4**	1.76 (0.99–3.14)	1.49 (0.85–2.60)	1.39 (0.79–2.43)	1.52 (0.87–2.67)	1.48 (0.83–2.66)
**SFA**					
**Q1**	1 (Ref)	1 (Ref)	1 (Ref)	1 (Ref)	1 (Ref)
**Q2**	1.88 (1.14–3.10)	1.78 (1.10–2.89)	1.81 (1.12–2.92)	1.81 (1.12–2.93)	1.87 (1.15–3.04)
**Q3**	3.18 (1.98–5.10)	3.22 (2.06–5.05)	3.24 (2.07–5.07)	3.25 (2.08–5.08)	3.45 (2.17–5.48)
**Q4**	3.68 (2.28–5.93)	3.48 (2.20–5.49)	3.50 (2.21–5.52)	3.55 (2.25–5.60)	4.02 (2.44–6.61)

† Model was adjusted for age, sex, ethnicity, BMI, waist circumference, Townsend deprivation index, education level, household income, self-reported smoking status, self-reported frequency of alcohol intake, physical activity, diet quality score, baseline hypertension, baseline diabetes,

baseline dyslipidemia, total cholesterol level, triglycerides level, total fatty acids level, serum ALT level, serum AST level, and blood platelet count.
‡ For n-3 PUFA and n-6 PUFA, rs174547 genotype was further adjusted for; for MUFA and SFA, rs102275 genotype was further adjusted for.
§ For n-3 PUFA, the remaining plasma fatty acids (n-6 PUFA, MUFA, SFA) were adjust for; for n-6 PUFA, the remaining plasma fatty acids (n-3 PUFA, MUFA, SFA) were adjust for; and so on. Total fatty acids level was not adjusted for here.

Supplementary Table 13. Sensitivity analyses of the HRs for the associations of plasma fatty acids levels with CLD mortality

Fatty acids	Exclude the first 2 years of follow-up †	Further adjusted for lipid-lowering medication	Further adjusted for PRS	Further adjusted for FADS1/2 genotype ‡	Further adjusted for remaining plasma fatty acids §
**n-3 PUFA**					
**Q1**	1 (Ref)	1 (Ref)	1 (Ref)	1 (Ref)	1 (Ref)
**Q2**	0.41 (0.28–0.59)	0.43 (0.30–0.60)	0.41 (0.30–0.58)	0.40 (0.28–0.57)	0.51 (0.37–0.73)
**Q3**	0.33 (0.22–0.50)	0.31 (0.21–0.45)	0.29 (0.20–0.43)	0.28 (0.19–0.41)	0.40 (0.27–0.58)
**Q4**	0.24 (0.15–0.39)	0.22 (0.14–0.35)	0.21 (0.13–0.33)	0.20 (0.12–0.32)	0.30 (0.19–0.47)
**n-6 PUFA**					
**Q1**	1 (Ref)	1 (Ref)	1 (Ref)	1 (Ref)	1 (Ref)
**Q2**	0.56 (0.37–0.85)	0.52 (0.35–0.76)	0.53 (0.36–0.77)	0.52 (0.35–0.76)	0.63 (0.42–0.93)
**Q3**	0.41 (0.24–0.69)	0.38 (0.24–0.61)	0.40 (0.25–0.64)	0.38 (0.24–0.62)	0.45 (0.27–0.74)
**Q4**	0.17 (0.08–0.35)	0.14 (0.07–0.28)	0.15 (0.08–0.30)	0.15 (0.08–0.29)	0.16 (0.08–0.33)
**MUFA**					
**Q1**	1 (Ref)	1 (Ref)	1 (Ref)	1 (Ref)	1 (Ref)
**Q2**	1.84 (1.07–3.15)	2.19 (1.33–3.60)	2.20 (1.33–3.63)	2.21 (1.34–3.65)	2.61 (1.56–4.36)
**Q3**	2.14 (1.22–3.75)	2.47 (1.47–4.16)	2.43 (1.44–4.09)	2.45 (1.46–4.12)	3.18 (1.85–5.45)
**Q4**	3.01 (1.51–6.00)	3.83 (2.04–7.21)	3.76 (2.00–7.08)	3.86 (2.05–7.25)	4.72 (2.44–9.13)
**SFA**					
**Q1**	1 (Ref)	1 (Ref)	1 (Ref)	1 (Ref)	1 (Ref)
**Q2**	1.24 (0.65–2.37)	1.30 (0.69–2.47)	1.32 (0.69–2.49)	1.32 (0.70–2.51)	1.10 (0.58–2.10)
**Q3**	1.79 (0.98–3.28)	1.87 (1.03–3.40)	1.88 (1.03–3.41)	1.90 (1.05–3.45)	1.49 (0.82–2.73)
**Q4**	5.03 (2.89–8.77)	6.16 (3.58–10.61)	6.31 (3.66–10.86)	6.36 (3.69–10.95)	4.42 (2.50–7.81)

† Model was adjusted for age, sex, ethnicity, BMI, waist circumference, Townsend deprivation index, education level, household income, self-reported smoking status, self-reported frequency of alcohol intake, physical activity, diet quality score, baseline hypertension, baseline diabetes,

baseline dyslipidemia, total cholesterol level, triglycerides level, total fatty acids level, serum ALT level, serum AST level, and blood platelet count.
‡ For n-3 PUFA and n-6 PUFA, rs174547 genotype was further adjusted for; for MUFA and SFA, rs102275 genotype was further adjusted for.
§ For n-3 PUFA, the remaining plasma fatty acids (n-6 PUFA, MUFA, SFA) were adjust for; for n-6 PUFA, the remaining plasma fatty acids (n-3 PUFA, MUFA, SFA) were adjust for; and so on. Total fatty acids level was not adjusted for here.

Supplementary Table 14. HR (95% CI) of HCC and CLD mortality for 1-SD increment of n-3 PUFA from subgroup analyses.

Subgroups	HCC		$P_{interaction}$	CLD mortality		$P_{interaction}$
	$HR_{1\text{-}SD}$ (95% CI)	*P* value		$HR_{1\text{-}SD}$ (95% CI)	*P* value	
**Age**			0.034			0.041
**<60 y**	0.55 (0.41–0.74)	<0.001		0.61 (0.48–0.77)	<0.001	
**≥60 y**	0.79 (0.67–0.93)	0.005		0.40 (0.31–0.52)	<0.001	
**Sex**			0.234			0.629
**Men**	0.76 (0.56–1.03)	0.076		0.52 (0.37–0.73)	<0.001	
**Women**	0.69 (0.58–0.82)	<0.001		0.51 (0.41–0.63)	<0.001	
**BMI**			0.540			0.531
**<25 kg/m^2**	0.82 (0.60–1.12)	0.214		0.61 (0.42–0.88)	0.009	
**≥25 kg/m^2**	0.69 (0.58–0.82)	<0.001		0.47 (0.38–0.58)	<0.001	
**Ethnicity**			0.346			0.253
**Others**	0.75 (0.39–1.45)	0.390		0.73 (0.37–1.42)	0.348	
**White**	0.72 (0.62–0.84)	<0.001		0.49 (0.40–0.59)	<0.001	
**Household income**			0.076			0.510
**<31000 £/y**	0.64 (0.52–0.79)	<0.001		0.44 (0.35–0.56)	<0.001	
**≥31000 £/y**	0.86 (0.66–1.10)	0.223		0.47 (0.33–0.69)	<0.001	
**Townsend index**			0.463			0.010
**Below median**	0.73 (0.59–0.90)	0.003		0.37 (0.26–0.53)	<0.001	
**Above median**	0.69 (0.56–0.86)	0.001		0.56 (0.45–0.69)	<0.001	
**Education level**			0.204			0.052
**Others**	0.78 (0.66–0.92)	0.003		0.55 (0.44–0.67)	<0.001	
**College**	0.59 (0.42–0.81)	0.001		0.44 (0.30–0.65)	<0.001	
**Smoking**			0.854			0.194
**Never**	0.73 (0.61–0.88)	0.001		0.56 (0.45–0.69)	<0.001	
**Current or previous**	0.67 (0.53–0.86)	0.001		0.43 (0.31–0.59)	<0.001	
**Alcohol drinking**			0.220			0.742

**Below monthly**	0.59 (0.44–0.78)	<0.001		0.47 (0.32–0.70)	<0.001	
**Weekly or daily**	0.77 (0.65–0.92)	0.003		0.51 (0.41–0.62)	<0.001	
**Activity**			0.441			0.658
**Below median**	0.75 (0.62–0.90)	0.003		0.50 (0.40–0.63)	<0.001	
**Above median**	0.63 (0.49–0.80)	<0.001		0.42 (0.32–0.56)	<0.001	
**Diet quality scores**			0.612			0.865
**<50**	0.75 (0.59–0.96)	0.022		0.49 (0.37–0.63)	<0.001	
**≥50**	0.66 (0.54–0.79)	<0.001		0.49 (0.38–0.64)	<0.001	
**Hypertension**			0.325			0.182
**No**	0.55 (0.39–0.78)	0.001		0.58 (0.39–0.85)	0.005	
**Yes**	0.76 (0.64–0.89)	0.001		0.45 (0.37–0.55)	<0.001	
**Diabetes**			0.688			0.342
**No**	0.73 (0.61–0.88)	0.001		0.51 (0.42–0.63)	<0.001	
**Yes**	0.67 (0.51–0.87)	0.003		0.43 (0.29–0.64)	<0.001	

Multivariable Cox proportional hazard model was used. Model was adjusted for age, sex, ethnicity, BMI, waist circumference, Townsend deprivation index, education level, household income, self-reported smoking status, self-reported frequency of alcohol intake, physical activity, diet quality score, baseline hypertension, baseline diabetes, baseline dyslipidemia, total cholesterol level, triglycerides level, total fatty acids level, serum ALT level, serum AST level, and blood platelet count.

Supplementary Table 15. HR (95% CI) of HCC and CLD mortality for 1-SD increment of n-6 PUFA from subgroup analyses.

Subgroups	HCC		$P_{interaction}$	CLD mortality		$P_{interaction}$
	HR_{1-SD} (95% CI)	*P* value		HR_{1-SD} (95% CI)	*P* value	
**Age**			0.003			0.882
**<60 y**	0.73 (0.49–1.11)	0.139		0.27 (0.20–0.37)	<0.001	
**≥60 y**	0.63 (0.47–0.84)	0.002		0.37 (0.24–0.56)	<0.001	
**Sex**			0.167			0.001
**Men**	0.73 (0.42–1.26)	0.259		0.17 (0.10–0.29)	<0.001	
**Women**	0.68 (0.52–0.88)	0.003		0.38 (0.28–0.50)	<0.001	
**BMI**			0.282			<0.001
**<25 kg/m^2**	0.66 (0.38–1.16)	0.147		0.17 (0.10–0.30)	<0.001	
**≥25 kg/m^2**	0.65 (0.50–0.85)	0.001		0.37 (0.27–0.49)	<0.001	
**Ethnicity**			0.588			0.350
**Others**	1.29 (0.50–3.29)	0.602		0.31 (0.10–0.98)	0.046	
**White**	0.64 (0.50–0.82)	<0.001		0.31 (0.23–0.40)	<0.001	
**Household income**			0.747			0.458
**<31000 £/y**	0.75 (0.55–1.03)	0.079		0.31 (0.22–0.44)	<0.001	
**≥31000 £/y**	0.60 (0.38–0.93)	0.022		0.30 (0.17–0.52)	<0.001	
**Townsend index**			0.184			0.446
**Below median**	0.62 (0.44–0.87)	0.005		0.26 (0.15–0.42)	<0.001	
**Above median**	0.73 (0.53–1.02)	0.063		0.31 (0.23–0.42)	<0.001	
**Education level**			0.568			0.737
**Others**	0.63 (0.48–0.82)	0.001		0.29 (0.22–0.39)	<0.001	
**College**	0.79 (0.48–1.30)	0.349		0.35 (0.20–0.61)	<0.001	
**Smoking**			0.953			0.430
**Never**	0.81 (0.60–1.09)	0.156		0.33 (0.24–0.46)	<0.001	
**Current or previous**	0.51 (0.35–0.75)	0.001		0.28 (0.18–0.43)	<0.001	
**Alcohol drinking**			0.088			<0.001

**Below monthly**	0.71 (0.48–1.05)	0.087		0.52 (0.31–0.89)	0.017	
**Weekly or daily**	0.67 (0.50–0.90)	0.008		0.24 (0.18–0.32)	<0.001	
**Activity**			0.134			0.077
**Below median**	0.58 (0.43–0.77)	<0.001		0.28 (0.21–0.39)	<0.001	
**Above median**	0.89 (0.60–1.31)	0.543		0.32 (0.20–0.50)	<0.001	
**Diet quality scores**			0.281			0.075
**<50**	0.72 (0.51–1.01)	0.060		0.33 (0.23–0.46)	<0.001	
**≥50**	0.66 (0.48–0.91)	0.011		0.27 (0.18–0.40)	<0.001	
**Hypertension**			0.092			0.548
**No**	0.75 (0.46–1.23)	0.258		0.40 (0.24–0.67)	0.001	
**Yes**	0.64 (0.49–0.83)	0.001		0.28 (0.21–0.39)	<0.001	
**Diabetes**			0.539			0.315
**No**	0.66 (0.49–0.89)	0.006		0.29 (0.21–0.38)	<0.001	
**Yes**	0.73 (0.49–1.07)	0.103		0.39 (0.22–0.69)	0.001	

Multivariable Cox proportional hazard model was used. Model was adjusted for age, sex, ethnicity, BMI, waist circumference, Townsend deprivation index, education level, household income, self-reported smoking status, self-reported frequency of alcohol intake, physical activity, diet quality score, baseline hypertension, baseline diabetes, baseline dyslipidemia, total cholesterol level, triglycerides level, total fatty acids level, serum ALT level, serum AST level, and blood platelet count.

Supplementary Table 16. HR (95% CI) of HCC and CLD mortality for 1-SD increment of MUFA from subgroup analyses.

Subgroups	HCC		$P_{interaction}$	CLD mortality		$P_{interaction}$
	HR_{1-SD} (95% CI)	*P* value		HR_{1-SD} (95% CI)	*P* value	
**Age**			0.003			0.318
**<60 y**	1.03 (0.69–1.54)	0.893		2.22 (1.59–3.10)	<0.001	
**≥60 y**	1.35 (1.02–1.80)	0.039		2.43 (1.62–3.65)	<0.001	
**Sex**			0.163			0.006
**Men**	1.31 (0.77–2.24)	0.316		2.44 (1.48–4.00)	<0.001	
**Women**	1.27 (0.98–1.64)	0.077		2.12 (1.57–2.88)	<0.001	
**BMI**			0.856			0.020
**<25 kg/m^2**	1.55 (0.89–2.71)	0.123		2.68 (1.53–4.72)	0.001	
**≥25 kg/m^2**	1.19 (0.92–1.54)	0.183		2.21 (1.65–2.96)	<0.001	
**Ethnicity**			0.579			0.349
**Others**	1.44 (0.46–4.55)	0.536		2.22 (0.61–8.05)	0.224	
**White**	1.21 (0.95–1.54)	0.118		2.30 (1.76–3.00)	<0.001	
**Household income**			0.327			0.288
**<31000 £/y**	1.31 (0.95–1.79)	0.098		2.99 (2.16–4.15)	<0.001	
**≥31000 £/y**	1.16 (0.75–1.81)	0.509		1.80 (1.04–3.12)	0.035	
**Townsend index**			0.051			0.239
**Below median**	1.52 (1.08–2.14)	0.017		3.14 (1.88–5.24)	<0.001	
**Above median**	1.05 (0.76–1.44)	0.772		2.15 (1.59–2.90)	<0.001	
**Education level**			0.680			0.102
**Others**	1.09 (0.84–1.42)	0.501		2.04 (1.52–2.74)	<0.001	
**College**	1.67 (0.98–2.85)	0.058		2.60 (1.50–4.51)	0.001	
**Smoking**			0.474			0.490
**Never**	0.92 (0.69–1.23)	0.572		2.02 (1.47–2.77)	<0.001	
**Current or previous**	1.91 (1.29–2.83)	0.001		2.45 (1.57–3.84)	<0.001	
**Alcohol drinking**			0.167			0.002

**Below monthly**	1.25 (0.85–1.85)	0.264		1.47 (0.85–2.53)	0.167	
**Weekly or daily**	1.22 (0.92–1.64)	0.174		2.72 (2.03–3.65)	<0.001	
**Activity**			0.247			0.057
**Below median**	1.25 (0.94–1.66)	0.129		2.12 (1.53–2.94)	<0.001	
**Above median**	1.22 (0.81–1.82)	0.339		2.67 (1.78–4.01)	<0.001	
**Diet quality scores**			0.718			0.296
**<50**	0.95 (0.67–1.34)	0.755		2.24 (1.57–3.18)	<0.001	
**≥50**	1.49 (1.09–2.05)	0.014		2.48 (1.70–3.62)	<0.001	
**Hypertension**			0.151			0.393
**No**	1.26 (0.75–2.13)	0.382		1.76 (0.98–3.16)	0.060	
**Yes**	1.25 (0.97–1.63)	0.090		2.57 (1.93–3.42)	<0.001	
**Diabetes**			0.586			0.044
**No**	1.33 (0.98–1.81)	0.071		2.59 (1.92–3.48)	<0.001	
**Yes**	1.11 (0.77–1.60)	0.582		1.78 (1.05–3.02)	0.032	

Multivariable Cox proportional hazard model was used. Model was adjusted for age, sex, ethnicity, BMI, waist circumference, Townsend deprivation index, education level, household income, self-reported smoking status, self-reported frequency of alcohol intake, physical activity, diet quality score, baseline hypertension, baseline diabetes, baseline dyslipidemia, total cholesterol level, triglycerides level, total fatty acids level, serum ALT level, serum AST level, and blood platelet count.

Supplementary Table 17. HR (95% CI) of HCC and CLD mortality for 1-SD increment of SFA from subgroup analyses.

Subgroups	HCC		$P_{interaction}$	CLD mortality		$P_{interaction}$
	$HR_{1\text{-}SD}$ (95% CI)	*P* value		$HR_{1\text{-}SD}$ (95% CI)	*P* value	
**Age**			0.580			0.596
**<60 y**	1.75 (1.38–2.22)	<0.001		2.07 (1.73–2.49)	<0.001	
**≥60 y**	1.39 (1.19–1.63)	<0.001		2.46 (1.96–3.09)	<0.001	
**Sex**			0.232			0.002
**Men**	1.40 (1.01–1.92)	0.041		3.08 (2.26–4.21)	<0.001	
**Women**	1.47 (1.27–1.69)	<0.001		1.98 (1.68–2.33)	<0.001	
**BMI**			0.088			0.101
**<25 kg/m²**	1.27 (0.92–1.74)	0.148		2.41 (1.80–3.21)	<0.001	
**≥25 kg/m²**	1.53 (1.33–1.77)	<0.001		2.04 (1.72–2.41)	<0.001	
**Ethnicity**			0.747			0.923
**Others**	0.90 (0.47–1.71)	0.736		2.68 (1.19–6.04)	0.017	
**White**	1.50 (1.31–1.72)	<0.001		2.15 (1.86–2.50)	<0.001	
**Household income**			0.530			0.100
**<31000 £/y**	1.46 (1.22–1.74)	<0.001		2.03 (1.68–2.46)	<0.001	
**≥31000 £/y**	1.41 (1.11–1.79)	0.004		2.30 (1.73–3.06)	<0.001	
**Townsend index**			0.572			0.122
**Below median**	1.40 (1.16–1.69)	<0.001		2.51 (1.91–3.29)	<0.001	
**Above median**	1.53 (1.27–1.83)	<0.001		2.08 (1.75–2.46)	<0.001	
**Education level**			0.331			0.542
**Others**	1.52 (1.31–1.76)	<0.001		2.27 (1.92–2.67)	<0.001	
**College**	1.42 (1.06–1.89)	0.019		2.05 (1.51–2.77)	<0.001	
**Smoking**			0.513			0.088
**Never**	1.47 (1.25–1.74)	<0.001		1.99 (1.67–2.37)	<0.001	
**Current or previous**	1.55 (1.25–1.93)	<0.001		2.78 (2.13–3.63)	<0.001	
**Alcohol drinking**			0.613			0.333

**Below monthly**	1.58 (1.26–1.99)	<0.001		2.15 (1.56–2.98)	<0.001	
**Weekly or daily**	1.41 (1.20–1.66)	<0.001		2.18 (1.86–2.57)	<0.001	
**Activity**			0.634			0.489
**Below median**	1.53 (1.31–1.80)	<0.001		2.39 (2.00–2.87)	<0.001	
**Above median**	1.42 (1.14–1.78)	0.002		2.10 (1.66–2.67)	<0.001	
**Diet quality scores**			0.377			0.509
**<50**	1.45 (1.19–1.75)	<0.001		2.10 (1.72–2.56)	<0.001	
**≥50**	1.53 (1.28–1.83)	<0.001		2.30 (1.87–2.85)	<0.001	
**Hypertension**			0.737			0.819
**No**	1.66 (1.24–2.22)	0.001		2.09 (1.53–2.86)	<0.001	
**Yes**	1.44 (1.25–1.67)	<0.001		2.17 (1.84–2.55)	<0.001	
**Diabetes**			0.657			0.087
**No**	1.46 (1.24–1.73)	<0.001		2.13 (1.81–2.50)	<0.001	
**Yes**	1.49 (1.20–1.86)	<0.001		2.17 (1.58–2.98)	<0.001	

Multivariable Cox proportional hazard model was used. Model was adjusted for age, sex, ethnicity, BMI, waist circumference, Townsend deprivation index, education level, household income, self-reported smoking status, self-reported frequency of alcohol intake, physical activity, diet quality score, baseline hypertension, baseline diabetes, baseline dyslipidemia, total cholesterol level, triglycerides level, total fatty acids level, serum ALT level, serum AST level, and blood platelet count.
