# Supplementary material for: Circulating fatty acids and risk of hepatocellular carcinoma and chronic liver disease mortality in the UK Biobank
Source: Nat Commun. 2024 May 2;15:3707. doi: 10.1038/s41467-024-47960-8 (PMC11065883; doi:10.1038/s41467-024-47960-8)
Supplement: Supplementary file 3 — Reporting Summary [file 41467_2024_47960_MOESM3_ESM.pdf]

nature portfolio

Corresponding author(s): Chengfu Xu

Last updated by author(s): Feb 26, 2024

Reporting Summary

Nature Portfolio wishes to improve the reproducibility of the work that we publish. This form provides structure for consistency and transparency in reporting. For further information on Nature Portfolio policies, see our Editorial Policies and the Editorial Policy Checklist.

Statistics

For all statistical analyses, confirm that the following items are present in the figure legend, table legend, main text, or Methods section.

n/a	Confirmed	
☐	☒	The exact sample size (*n*) for each experimental group/condition, given as a discrete number and unit of measurement
☐	☒	A statement on whether measurements were taken from distinct samples or whether the same sample was measured repeatedly
☐	☒	The statistical test(s) used AND whether they are one- or two-sided<br>*Only common tests should be described solely by name; describe more complex techniques in the Methods section.*
☐	☒	A description of all covariates tested
☐	☒	A description of any assumptions or corrections, such as tests of normality and adjustment for multiple comparisons
☐	☒	A full description of the statistical parameters including central tendency (e.g. means) or other basic estimates (e.g. regression coefficient) AND variation (e.g. standard deviation) or associated estimates of uncertainty (e.g. confidence intervals)
☐	☒	For null hypothesis testing, the test statistic (e.g. *F*, *t*, *r*) with confidence intervals, effect sizes, degrees of freedom and *P* value noted<br>*Give P values as exact values whenever suitable.*
☒	☐	For Bayesian analysis, information on the choice of priors and Markov chain Monte Carlo settings
☒	☐	For hierarchical and complex designs, identification of the appropriate level for tests and full reporting of outcomes
☐	☒	Estimates of effect sizes (e.g. Cohen's *d*, Pearson's *r*), indicating how they were calculated

Our web collection on statistics for biologists contains articles on many of the points above.

Software and code

Policy information about availability of computer code

Data collection	No software was used.
Data analysis	SAS 9.4 (SAS Institute Inc, Cary, NC). SAS codes for the analyses are available at https://github.com/liuznCHN/UKB

For manuscripts utilizing custom algorithms or software that are central to the research but not yet described in published literature, software must be made available to editors and reviewers. We strongly encourage code deposition in a community repository (e.g. GitHub). See the Nature Portfolio guidelines for submitting code & software for further information.

Data

Policy information about availability of data

All manuscripts must include a data availability statement. This statement should provide the following information, where applicable:

- Accession codes, unique identifiers, or web links for publicly available datasets
- A description of any restrictions on data availability
- For clinical datasets or third party data, please ensure that the statement adheres to our policy

The datasets analyzed during the current study are available in the UK Biobank Repository. This research has been conducted using the UK Biobank resource under application number 79302.

Research involving human participants, their data, or biological material

Policy information about studies with human participants or human data. See also policy information about sex, gender (identity/presentation), and sexual orientation and race, ethnicity and racism.

Reporting on sex and gender	Analyses are adjusted for sex.
Reporting on race, ethnicity, or other socially relevant groupings	The main analyses are adjusted for ethnicity.<br>Genetic analysis was restricted to participants with White descent.
Population characteristics	The characteristics of all participants by quartiles of plasma FA are summarized in Table 1.
Recruitment	Recruitment of study participants in the UK Biobank is based on volunteer enrollment during 2006–10 and their health is being followed long term.
Ethics oversight	The UK Biobank received ethics approval from the North West Multi-Center Research Ethics Committee (REC No. 16/NW/0274).

Note that full information on the approval of the study protocol must also be provided in the manuscript.

Field-specific reporting

Please select the one below that is the best fit for your research. If you are not sure, read the appropriate sections before making your selection.

☐ Life sciences ☒ Behavioural & social sciences ☐ Ecological, evolutionary & environmental sciences

For a reference copy of the document with all sections, see nature.com/documents/nr-reporting-summary-flat.pdf

Behavioural & social sciences study design

All studies must disclose on these points even when the disclosure is negative.

Study description	The UK Biobank is a large prospective study in the UK to investigate the role of genetic factors, environmental exposures and lifestyle in the causes of major diseases of late and middle age. Here, we aim to explore the associations of plasma fatty acids with the risk of hepatocellular carcinoma (HCC) and chronic liver disease (CLD) mortality.
Research sample	This research is based on an existing dataset. The UK Biobank is a community-based cohort of 502,506 volunteers aged 39 to 73 years across England, Scotland and Wales, but not well representative of the sampling population (https://doi.org/10.1093/aje/kwx246). The current project included 274,123 participants with valid data on NMR metabolic biomarkers. We then excluded those with end-stage liver diseases (Supplementary Table S1), unavailable genetic or FIB-4 data, and missing covariates, leaving 252,398 participants to analyze the associations. In addition, a subgroup of participants of European descent with available genetic data (n=236,492) was extracted only for genetic analyses (Supplementary Figure S1).
Sampling strategy	UK Biobank investigators sent postal invitations to 9,238,453 individuals registered with the UK's National Health Service who lived within approximately 25 miles of one of 22 assessment centers located throughout England, Wales, and Scotland. UK Biobank also received approval to retain limited information on nonresponders. Overall, 503,317 participants consented to join the study cohort and visited an assessment center between 2006 and 2010, resulting in a participation rate of 5.45% (https://doi.org/10.1093/aje/kwx246).
Data collection	At a baseline visit, participants completed a face-to-face interview, a touch screen questionnaire, physical examinations and provided biological samples after providing written informed consent. The measurements of metabolic biomarkers took place between June 2019 -April 2020 (phase 1) and April 2020 - June 2022 (phase 2) using a high-throughput nuclear magnetic resonance (NMR)-based profiling platform (Nightingale Health Ltd.). Altogether 251 metabolic biomarkers (including lipoprotein lipids in 14 subclasses, fatty acids and fatty acid compositions, as well as various low-molecular weight metabolites) for EDTA plasma samples (collected at baseline) were measured from a randomly selected subset of approx 280,000 UK Biobank participants. Given that this is a population-level observational study, blinding is not available.
Timing	Follow-up time in person-years was calculated from the date of attendance (2006-2010) until the date of HCC diagnosis, death, or the end of follow-up (19 December 2022).
Data exclusions	The current project included 274,123 participants with valid data on NMR metabolic biomarkers. We then excluded those with end-stage liver diseases (Supplementary Table S1), unavailable genetic or FIB-4 data, and missing covariates, leaving 252,398 participants to analyze the associations. In addition, a subgroup of participants of European descent with available genetic data (n=236,492) was extracted only for genetic analyses (Supplementary Figure S1).
Non-participation	61 participants withdraw from the UK biobank.
Randomization	Given that this is a non-experimental study, randomization was not used.

Reporting for specific materials, systems and methods

We require information from authors about some types of materials, experimental systems and methods used in many studies. Here, indicate whether each material, system or method listed is relevant to your study. If you are not sure if a list item applies to your research, read the appropriate section before selecting a response.

Materials & experimental systems

n/a	Involved in the study
☒	☐ Antibodies
☒	☐ Eukaryotic cell lines
☒	☐ Palaeontology and archaeology
☒	☐ Animals and other organisms
☒	☐ Clinical data
☒	☐ Dual use research of concern
☒	☐ Plants

Methods

n/a	Involved in the study
☒	☐ ChIP-seq
☒	☐ Flow cytometry
☒	☐ MRI-based neuroimaging

Plants

Seed stocks	N/A
Novel plant genotypes	N/A
Authentication	N/A
